# A Solid State Nanopore Device for Investigating the Magnetic Properties of Magnetic Nanoparticles

**DOI:** 10.3390/s130606900

**Published:** 2013-05-24

**Authors:** SangYoon Park, Jaekwan Lim, Y. Eugene Pak, Seunghyun Moon, Yoon-Kyu Song

**Affiliations:** 1 Advanced Institutes of Convergence Technology, Suwon 443-270, Korea; E-Mail: jakelim09@gmail.com (J.L.); genepak@snu.ac.kr (Y.P.); 2 Department of Transdisciplinary Studies, Seoul National University, Seoul 151-742, Korea; E-Mail: irus7711@snu.ac.kr

**Keywords:** nanopore, magnetic nanoparticle, ion current blockade, magneto-susceptibility

## Abstract

In this study, we explored magnetic nanoparticles translocating through a nanopore in the presence of an inhomogeneous magnetic field. By detecting the ionic current blockade signals with a silicon nitride nanopore, we found that the translocation velocity that is driven by magnetic and hydrodynamic forces on a single magnetic nanoparticle can be accurately determined and is linearly proportional to the magnetization of the magnetic nanoparticle. Thus, we obtained the magneto-susceptibility of an individual nanoparticle and the average susceptibility over one hundred particles within a few minutes.

## 1. Introduction

The most important issue for magnetic materials is an understanding of the magnetic and physical properties of a nanometer-sized particle because the use of magnetic nanoparticles (MNPs) in many applications depends predominantly on their inherent magnetic properties. In the case of biomedical applications, the forces that influence the translational, rotational, and vibrational motion of a MNP- tagged biomolecule are the magnetic force due to the magnetization of an individual MNP and the external magnetic field under a given condition [1–3]. Popular magnetic biosensing platforms such as giant magneto-resistance (GMR) sensors, Hall sensors, and magneto-optical sensors that are used to quantitatively analyze the existence of target molecules are based on the detection of a stray field from a single MNP [4–7]. Furthermore, structural assemblies of MNPs form various recording media and permanent magnets [8].

Recently, there have been many attempts to quantitatively analyze a biomolecule by magnetically manipulating the motion of magnetic beads in a microfluidic system because magnetic interactions are generally not affected by changes in the surface charges, pH, or ionic concentration of the surrounding medium, contrary to electrical-driven manipulation [9].

To precisely manipulate the motion, we should know the characteristics of the MNP, e.g., the size, charge state, and magnetism. There are well-established techniques for investigating the physical and electrical properties of a nanoparticle. For instance, the size and surface charge state of a nanoparticle can be routinely characterized by dynamic light scattering (DLS) and zeta-potentiometry, respectively. These methods can provide information on the distributions and the average values of the size and charge number of each particle. Magnetic characteristics have been usually determined by a superconducting quantum interference device (SQUID) and by a vibrating sample magnetometer (VSM). Unlike DLS and zeta-potentiometry, these techniques, although they have excellent sensitivities, are limited to the average properties of a large number of MNPs. To the best of our knowledge, the resultant data from these techniques do not provide sufficient information on the magnetism of a MNP, which may be considered as a critical bottleneck in facilitating the use of MNPs in various industrial applications. Therefore, the need to characterize a single MNP has been increasingly recognized.

In this work, we investigate the motion of MNPs, 30 nm in diameter, that are driven by both magnetic and electric forces in a nanopore membrane. In this investigation, we measured the velocity of the MNPs passing through the nanopore using an ionic current blockade. We found that the magnetic force enables the MNPs to move more rapidly and that the velocity is linearly proportional to the magnetization of a MNP. Thus, we were able to measure the magnetization of a single colloidal MNP and to acquire hundreds of data points on the magnetization within a few minutes.

## 2. Methods

**Figure 1.**
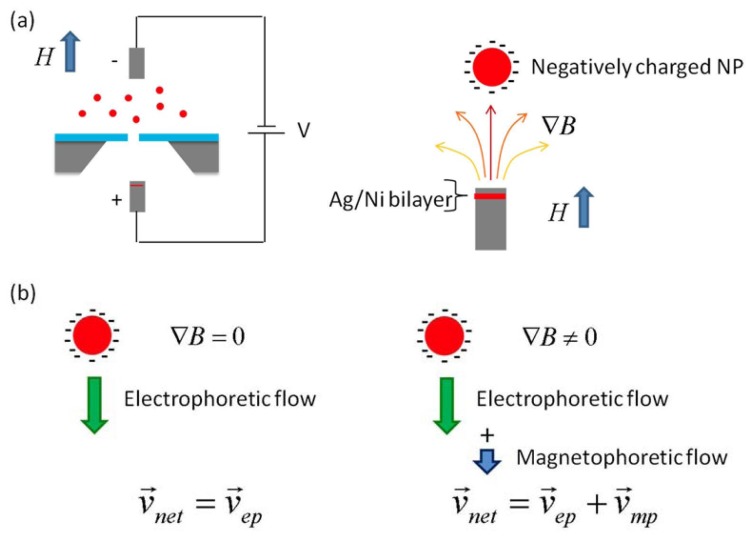
Schematic of the nanopore device that a single magnetic nanoparticle passes through; (**a**) a charged magnetic NP in solution moves through the nanopore under both an electric field (electrophoresis) and a magnetic field gradient (magnetophoresis) (**b**) comparison of the net velocity vector of a charged magnetic NP due to an electric field (left) and to both electric and magnetic fields (right).

A nanopore chip, which has a single nanometer-sized hole in a free-standing ultrathin membrane between two miniaturized fluidic chambers (“*cis*” and “*trans*”), is hydrated by conducting a fluid through it. An electric potential is applied across the chip; an electric current is used because the conduction of ions through the nanopore can be observed. When MNPs with negative charges are immersed in the *cis* chamber, they are captured and linearly threaded through the nanopore from the *cis* to *trans* chamber, as shown in Figure 1, and the current between the two electrodes is momentarily interrupted, which is the so-called “ionic current blockade”. The magnitude (ΔIc) and dwell-time (Δt) of the current blockade are governed by the size of the particle under given conditions [10]:

(1) $$\Delta I_{C}=\left[S\;(d_{C}\;,d_{S}\;)\frac{{d_{S}}^{3}}{\;(l_{C}+0.8d_{C}\;){d_{C}}^{2}}\right]I_{C}$$

(2) $$\Delta t=\frac{2\pi \eta {\;(I_{C}+0.8d_{C}\;)}^{2}d_{S}\;(1+d_{S}/2d_{D}\;)}{VQ}$$

(3) $$v_{ep}\equiv \frac{l_{C}}{\Delta t}=l_{C}\;\left[\frac{2\pi \eta {\;(I_{C}+0.8d_{C}\;)}^{2}d_{S}\;(1+d_{S}/2d_{D}\;)}{VQ}\right]$$

Here, Ic is the measured ionic current upon the application of a membrane potential (V), and Q is the charge on a particle; lc, dc, dd, and ds are the channel length, channel width, Debye length and particle diameter, respectively. For the simplicity, the counter flow effect induced by the counter-ion buildup around the charged nanoparticle was not included. The factor 0.8dc corrects for the so-called “end effect”, which becomes significant when the pore diameter is comparable to the pore length [8]. S(dc, ds) is a correction factor that depends on the relative values of dc and ds, and is very close to 1 for most cases [11]. From the measured current (ΔIc) and dwell-time (Δt), we determine not only the diameter of a single particle but also the translocation velocity, which is mainly due to electrophoresis in the nanopore.

By applying an inhomogeneous magnetic field parallel to the electric field, the MNPs can be pulled in the field direction due to a magnetophoretic flow, which can be simply described by the relation between the magnetic drag force (F_mag_) and the opposing hydrodynamic drag force (Fh) [12,13]:

(4) $${\vec{F}}_{H}={\vec{F}}_{\text{mag}}$$

(5) $$6\pi \eta v_{mp}\cdot \frac{d_{S}}{2}=\frac{V\chi }{\mu_{0}}\;(\vec{B}\cdot \nabla \;)\vec{B}=\frac{\pi \cdot {d_{S}}^{3}\cdot \chi }{6\cdot \mu_{0}}\;(\vec{B}\cdot \nabla \;)\vec{B}$$

(6) $$v_{mp}=\frac{{d_{S}}^{2}\chi }{18\eta \mu_{0}}\;(\vec{B}\cdot \nabla \;)\vec{B}$$

Here, η is the viscosity of the surrounding medium and *v*_mp_ is the velocity of a particle due to the magnetophoretic flow. The resultant velocity is linearly proportional to the magnetization of a single MNP. Therefore, magnetization can be obtained by measuring the net velocity (*v*_net_), which is composed of *v*_mp_ and *v*_ep_.

The basic principle of work is based on the following two factors: (i) electrophoresis: the pull of the MNP into the nanopore with *v*_ep_; (ii) magnetophoresis: changing the net velocity (*v*_net_ = *v*_ep_ + *v*_mp_). The velocity obtained is converted into the magnetization per particle using Equation (6).

## 3. Experiments

**Figure 2.**
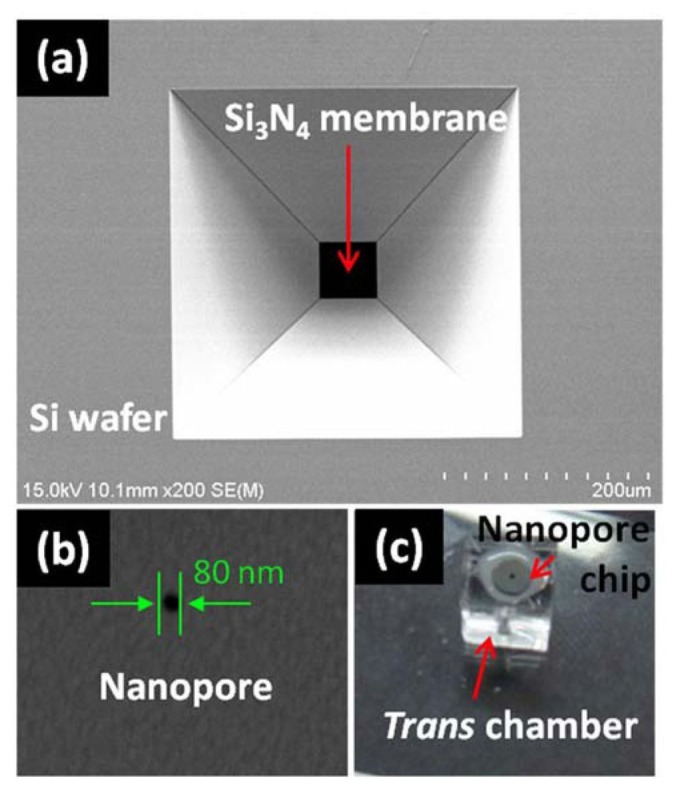
(**a**), (**b**) SEM images of a solid state nanopore (Si_3_N_4_ membrane on a Si wafer) which was fabricated by MEMS technology and then drilled by focused ion-beam (FIB) etching; (**c**) photographic view of the PDMS chamber configuration.

The detector that was used in this study was a solid-state nanopore, which was fabricated in a freestanding silicon nitride (Si_3_N_4_) membrane using conventional MEMS technology (see Figure 2) [14,15]. The Ic, and dc were kept at 30 nm and 80 nm, respectively. The *cis* and *trans* chamber set was fabricated with PDMS (Sylgard 184, Dow Corning, Inc., Midland, MI, USA) and was 1 mm in diameter. A 0.5 M KCl solution without buffer was used as a conducting solution and was prepared using deionized water and KCl powder purchased from Sigma-Aldrich, Co. (St. Louis, MO, USA), followed by filtration through a 0.2 syringe filter (Whatman, Ltd., Piscataway, NJ, USA) prior to use. The MNPs that were used in this experiment were obtained from Nanobrick, Inc. (Suwon, Korea) and from Micromod, GmbH (Rostock, Germany) and were negatively charged and tetrabutylammonium hydroxide-stabilized.

The Si_3_N_4_ nanopore was clamped between the two half chambers, sealed with epoxy, and then placed in the center of a vertical Helmholtz coil in a Faraday cage on a vibration-isolated optical bench. The ionic current was measured in the voltage-clamp mode with an integrated data acquisition system (Axopatch 200B and Digidata 1440A, Molecular Devices, LLC, Sunnyvale, CA, USA). An inhomogeneous magnetic field was generated around the nanopore by magnetic induction from a Ag/Ni/AgCl electrode coupled with the magnetic field in the Helmholtz coil. The magnetic field was measured using a commercial magnetic sensor (Model 410 Gaussmeter, Lake Shore Cryonics, Inc., Westerville, OH, USA) to obtain a spatial distribution of the magnetic field. First, the magnetic sensor was located at the fixed position where the nanopore would be located in the translocation velocity measurements. Then, the Ag/Ni/AgCl electrode was moved precisely from one location to another by a micromanipulator to measure a distance (between the nanopore and the Ni-coated electrode) dependent magnetic fields. Thus, the magnetic field profile near the nanopore was obtained at a given (induced) magnetic field, and then the gradient of the magnetic field at the nanopore was extracted from the measured magnetic field profile. With the information obtained above, we could magnified measure translocation velocity under various magnetic fields, and extract the magnetization-magnetic field (M-B) curve from Equation (6). The Ag/Ni/AgCl electrode was fabricated by e-beam evaporation of Ni and Ag on a AgCl pellet.

## 4. Results

**Figure 3.**
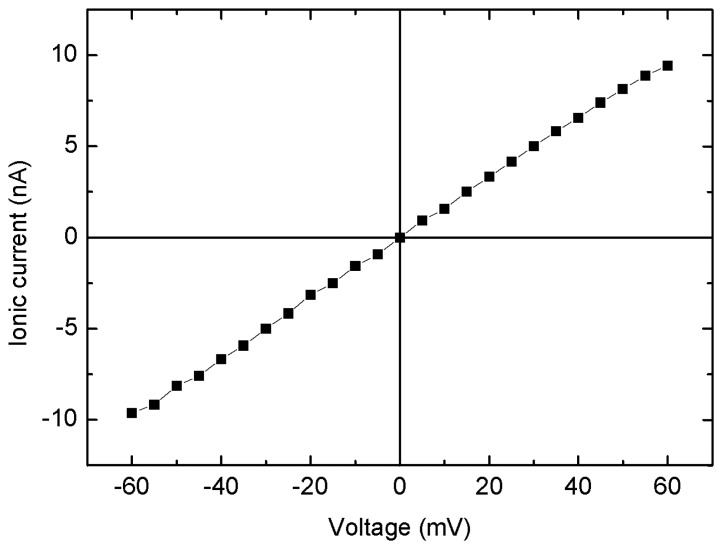
A measurement of the ionic current *vs.* applied voltage through the solid-state nanopore.

The Ic-V characteristics of the nanopore device with an 80 nm pore diameter and a 30 nm thick silicon nitride membrane are shown in Figure 3. The voltage was swept from −0.1 V to +0.1 V. The experimental and theoretical [16] conductance values were found to be 126 and 285 nS, respectively. Thus, the theoretical value (285 nS) is twice as large as the experimental value (126 nS). This inconsistency may be due to the underestimated dimension and the non-ideal shape of the nanopore. The calculation of the conductance used the assumption that the nanopore is cylindrical and has a very smooth wall. However, the actual shape is not cylindrical, but rather is close to hourglass-like [17,18]. Therefore, the effective length is shorter and the radius is smaller than the actual physical diameter which could be determined by FE-SEM. Furthermore, the roughness of the nanopore wall effectively reduces the diameter.

**Figure 4.**
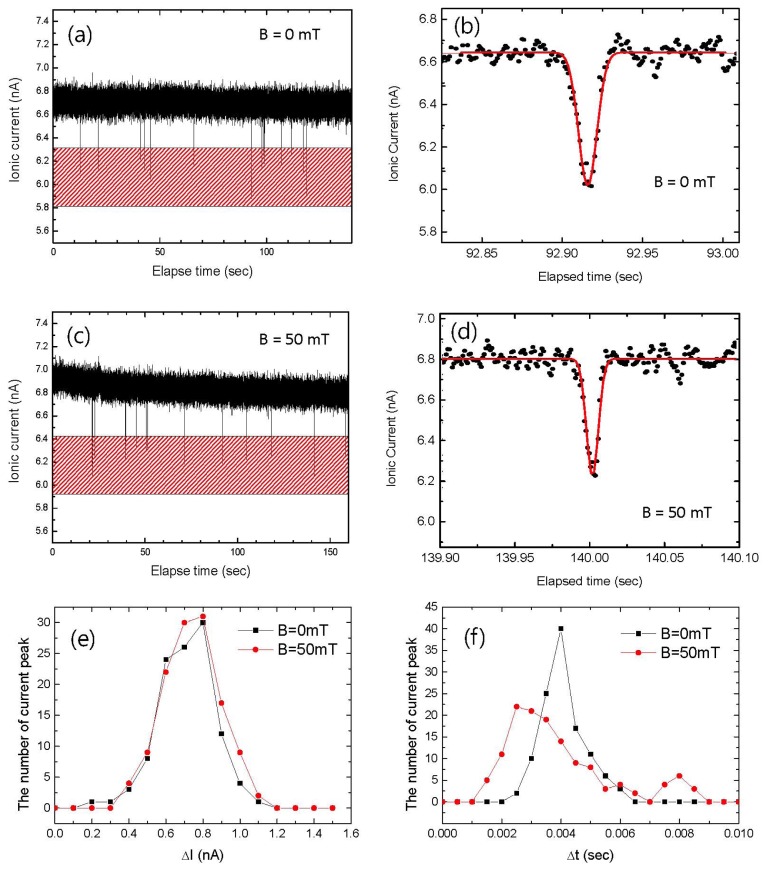
Time-dependent ionic current of charged magnetic NPs in the absence of magnetic field, (**a**) a global view and (**b**) a magnified view, and in the presence of a magnetic field, (**c**) a global view and (**d**) a magnified view. (**e**) Distribution of the ionic current blockade amplitude (ΔI) for 300 sec. (**f**) Distribution of the ionic current blockade dwell time (Δt).

Figure 4 shows the time-dependent ionic current (Ic *vs.* t recording) for 5 min. Hundreds of current blockade events could be observed due to MNP translocation for both B = 0 in Figure 4(a,b) and B ≠ 0 in Figure 4(c,d). The current blockades that are shown in Figure 4(b,d) have a slightly asymmetric triangular shape, consistent with those of spherical particles in previous works [19]. To obtain ΔIc and Δt, the current blockades were simply fitted with a Gaussian curve rather than with multiple peaks because Gaussian fitting is more intuitive for the understanding of the motion of a single sphere. By fitting a hundred current blockade events, histograms of ΔIc and Δt were obtained as shown in Figure 4(e,f), respectively. It should be noted that the average value of ΔIc for B = 0 mT was the same as that for B = 50 mT, whereas Δt for B = 50 mT was shortened by approximately 28%. The histograms reflect the fact that ΔIc is only dominantly governed by the size of a single particle from Equation (1), while Δt stems from the velocity that is driven not only by the electrophoresis from Equation (3) but also by the magnetophoresis from Equation (6). Therefore, the size and magnetization of a single MNP could be determined from the ΔIc and Δt histograms, respectively.

**Figure 5.**
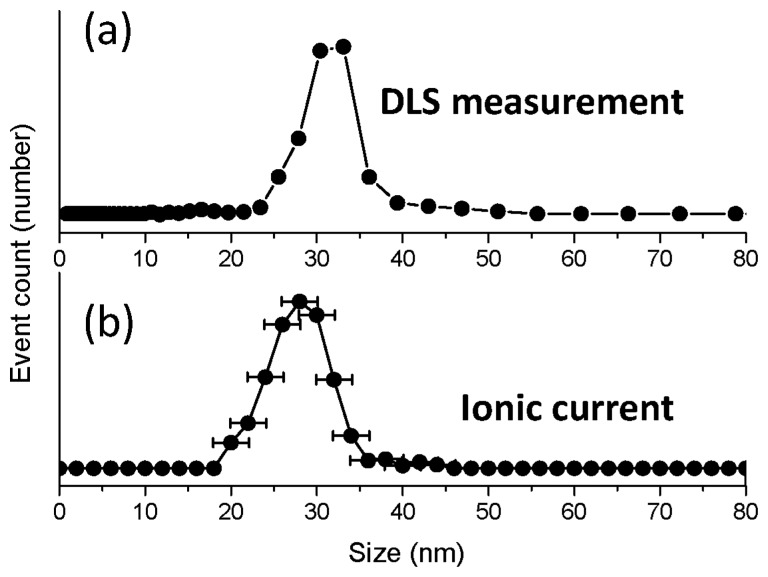
Size distribution of the magnetic nanoparticle obtained from (**a**) the dynamic light scattering (DLS) method and (**b**) the ionic current blockade.

We compared the size distribution from ΔIc with the one taken from dynamic light scattering (DLS) as shown in Figure 5. The distribution from the DLS experiment, which is shown in Figure 5(a), revealed that the most frequent size and the average value are 33 nm and 31.3 nm, respectively, which are very close to the anticipated numbers. The distribution from ΔIc, which is shown in Figure 5(b), was in a good agreement with that from DLS. The error likely arises from the background current noise.

**Figure 6.**
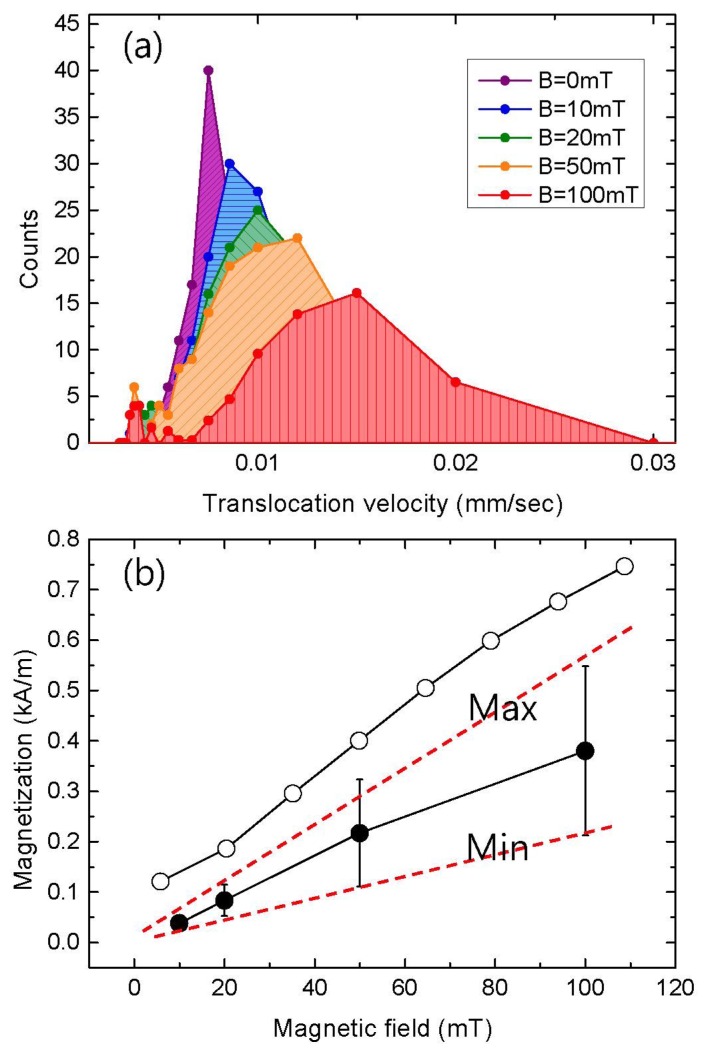
**(a)** Velocity distribution of the magnetic nanoparticles under various magnetic fields. (**b**) M-B curves obtained from VSM measurements (open circle) and ionic current measurements (closed circle).

To explicitly verify the effect of the magnetic force on the velocity, the translocation velocity was measured as a function of the external magnetic field, as shown in Figure 6(a). It can be seen that the velocity increased with increasing external magnetic field. This tendency indicates that the change in the velocity is mainly driven by the magnetic force between the external field and the MNPs. It should be noted that the histograms *broadened* with an increase in the magnetic field. The dependence of this broadening on the external magnetic field could be explained by the existence of slightly different magnetizations among the MNPs, which would lead to a variation in the slope of the M-B curve (which is proportional to the magnetic susceptibility). This variation in the susceptibility would enable the distribution of the velocity to broaden with an increase in the external field. Figure 6(b) shows a comparison of the M-B curves from the velocity measurements with that from VSM measurements. The velocity was converted to the magnetization per particle using Equation (6) in the Section 2. For high magnetic field regime at B = 100 mT, the gradient of the magnetic field on the order of 100 T/m was obtained from the field *vs.* distance measurement as described in the Section 3. The susceptibility from the ionic current was linearly proportional to the magnetic field in the range of B < 100 mT, and was approximately half the value of the VSM result of 1.6. The discrepancy in the absolute value of the susceptibility might be due to errors in the effective parameter estimations including the “end effect”, but is still under investigation.

## 5. Conclusions

We have investigated the properties of magnetic nanoparticles translocating through a solid-state nanopore in the presence of an inhomogeneous magnetic field. By measuring and analyzing the ionic current blockade, the translocation velocity of a nanoparticle passing through a nanometer-scale channel was found to be linearly proportional to the magnetization of the single magnetic nanoparticle. We have also shown the possibility of using this method to analyze the magnetic properties of hundreds of individual nanoparticles within a few minutes. We expect that this method will find a number of interesting applications, not only for characterizing the magnetic properties of inorganic nanoparticles but also for manipulating and analyzing organic and biological molecules that have been hybridized with magnetic nanoparticles.
